# The Impact of a 1-Year COVID-19 Extension on Undergraduate Dentistry in Dundee: Final Year Students’ Perspectives of Their Training in Oral Surgery

**DOI:** 10.3390/dj10120230

**Published:** 2022-12-06

**Authors:** Michaelina Macluskey, Angela S. Anderson, Simon D. Shepherd

**Affiliations:** Department of Oral Surgery, School of Dentistry, University of Dundee, Dundee DD1 4HN, UK

**Keywords:** education, oral surgery, COVID-19, dental student self-confidence, dental student opinion

## Abstract

Background: The detrimental impact of the COVID-19 pandemic on dental education prompted the Scottish Government to fund an additional year to the dental course to ensure that the students had the necessary clinical experience. The aim of the study was to better understand the final year student perceptions of this extension on their oral surgery experience at the University of Dundee. Methods: This mixed methods study consisted of an anonymous online questionnaire and a focus group. Results: Forty-one students (69.3%) completed the questionnaire and ten students participated in the focus group. Thirty-six (88.8%) students agreed that the oral surgery teaching provided sufficient knowledge to undertake independent practice. All of the students felt confident to carry out an extraction, and the majority of them (*n* = 40, 95%) felt confident to remove a retained root, however, their confidence with surgery was lower. Conclusion: The extension gave the students sufficient experience in oral surgery to gain confidence in clinical skills and an appropriate level of knowledge in preparation for the next phase of their career. Most of the students agreed that the extension was necessary and beneficial. This cohort graduated with more oral surgery experience than any of the students did in the previous 4 years from Dundee and with experience that was comparable with the students at other schools in the pre-COVID-19 era.

## 1. Introduction

The novel coronavirus SARS-CoV-2 (Severe Acute Respiratory Syndrome Coronavirus-2) caused an acute respiratory disease (coronavirus disease 2019, COVID-19) in the human population [1,2]. The United Kingdom (UK) experienced emergency pandemic restrictions with an imposed lockdown on March 2020 [3]. The impact of COVID-19 on dentistry was significant [4]. Clinical access restrictions were severe, only emergency or urgent attendances were permitted, with all of the elective treatment provisions being postponed. Personal protective equipment shortages further reduced the opportunities for clinical treatment, as did staff-related COVID-19 illness incidents [5].

The impact on undergraduate dental education was immense, and the immediate upheaval caused by the pandemic compelled dental schools to re-deploy efforts to prepare students for successful graduation [6,7,8,9,10,11,12,13]. Although alternative online teaching approaches might be well suited to didactic lecture-based materials, they are a poor substitute for clinical hands-on teaching [14]. Our final year undergraduate students attend oral surgery for a one-week block (OSB) which consists of teaching in consultant clinics, biopsy sessions, minor oral surgery (MOS) and access to general anaesthesia sessions for adult oral surgery, paediatric exodontia and oral and maxillofacial surgery. The students normally attend 10 sessions in semester one and 5 sessions in semester two, during which the students are required to complete a minimum of two MOS cases. This target is in keeping with other UK dental schools according to a UK-based survey [15]. Regardless of the targets for the completed cases, the primary goal remains that a student must be considered suitably prepared to exit dental school as a clinician who is not only personally ready but also considered capable of practicing clinical dentistry [16]. Understandably, reaching these numbers in pandemic conditions was significantly impeded. In Scotland, the three dental schools (Aberdeen, Dundee, and Glasgow) in close association and collaboration with the Board for Academic Dentistry, the Scottish Funding Council, NHS Education for Scotland, and Scottish Government were forced to consider appropriate contingencies. The conclusion was that all students in all of the years of the BDS programme in Dundee and Glasgow would be required to undertake a 1-year extension to reach the status of ‘safe beginner’ for registration with the General Dental Council [17]. The decision undoubtedly affected the students at many different levels, but there are reports on the adverse impact of COVID-19 on the dental students’ stress levels [14,18], and no accounts could be found on the impact on their clinical experience in comparison to that of the pre-COVID-19 levels or of other extensions to the dental course.

The primary aim of this study was to better understand our final year students’ perceptions of this one-year COVID-19 enforced extension on their clinical training experiences and oral surgery (OS) teaching. A secondary aim was to compare the oral surgery clinical experience of this cohort with that of the pre-COVID-19 cohorts to determine whether the extension allowed them to attain a similar clinical experience prior to graduation.

## 2. Materials and Methods

This mixed methods study consisted of an online student questionnaire and a student focus group. Ethical approval was granted by The University of Dundee Ethics Committee (UOD-SREC-SDEN-2022-002).

Part 1. Questionnaire

At the end of the academic year 2022, all final year dentals students (*n* = 60) were invited to complete a voluntary, anonymous online questionnaire. The questionnaire included their self-perceived confidence in OS, their experience during the extended year, the impact of the extended year, and their perceptions of the impact of COVID-19. The questions were answered in a combination of Likert responses on a scale of 1–5 [19], dichotomous responses, a selection of pre-determined response options, and free text. The questions in Section B on self-perceived confidence in oral surgery were the same as in a previously published national survey [20]. The survey link was sent to the 60 final year students’ university email addresses, and a reminder email was sent out 2 weeks later to encourage greater participation.

Part 2. Focus Group

Once the online questionnaire had been completed, to better understand the responses and add richness to the evaluation of this teaching, the students were invited to join a qualitative evaluation focus group. An email invitation, along with participation information, was distributed to the entire year group, inviting those students who had completed the survey to attend the focus group session. The students confirmed their involvement and consent by sending a return email. It was planned that we would recruit a maximum of 10 participants to the focus group. The participants attended remotely using Microsoft Teams on 10 May 2022. The discussion was led by us asking a set of pre-determined questions (Table 1). The proceedings were audio recorded, transcribed, and checked for accuracy. A thematic analysis of the transcribed discussion was carried out by the three authors independently using an inductive approach. The final qualitative account was achieved via a consensus being reached following the discussion. The results were shared with two participants from the focus group to determine whether the interpretation of the data was consistent with their views.

Part 3. Comparison with previous final year student activity

At the University of Dundee, the student clinical activity was recorded using the LiftUpp Software which is an i-Pad based assessment and a student development tool [21,22]. To determine whether this cohort of students achieved a similar experience to the previous pre-COVID-19 cohorts, their data were compared for the number of extractions and MOS cases.

A comparison of their self-perceived confidence was performed by correlating the responses to Section B of the questionnaire with an identical question set from a previous UK-wide study [20] which also included final year students from the University of Dundee. The comparisons of the clinical experience data were performed using a one-way analysis of variance (ANOVA) with a 95% confidence interval. This was followed by multiple post hoc comparisons using a Least Significance Test to determine exactly where there was a significant difference between the 2022 cohort for the mean number of extractions or surgical cases and the cohorts from 2017, 2018, 2019, and 2020. Correlations between the extended year cohort and the national data [20] were performed using Spearman’s correlation coefficient. The data were analysed by using SPSS Statistics for Windows (IBM SPSS Statistics for Windows, Version 23.0. IBM Corp: Armonk, NY, USA).

## 3. Results

### 3.1. Questionnaire

Forty-one students responded to the questionnaire (69.3%), of which 27 (65.9%) were female. Six students (14.6%) were graduate entry students with one student having a health care qualification (2.4%).

The responses to Section B of the questionnaire are shown in Table 2. The majority (36 students 88.8%), either agreed or strongly agreed that the OS teaching provided sufficient knowledge to undertake independent practice, and 100% felt confident to undertake an extraction. Their confidence in the removal of a visible retained upper molar root was high, with 21 (51.2%) of them responding with strongly agree and 18 (43.9%) of them responding with agree, however this gradually fell with the descriptions of more difficult procedures. The confidence in raising a mucoperiosteal flap and bone removal was lower, with 30 students responding negatively (73%) and only 5 students (12.2%) agreed that they felt confident to section a tooth in the process of surgery. The confidence in their ability to suture flaps was high, with 5 (12.2%) of them strongly agreeing and 33 (80.5%) of them agreeing that they felt confident to place a suture. The free text responses indicated that they would have liked to obtain more surgical experience:

Examples:

*“I feel that there should be additional OSB throughout the year and would be ideal to have been able to participate in additional MOS as although I felt capable under supervision, I feel nervous about completing this treatment on my own. I feel, in particular, cutting flaps in aesthetic regions and bone removal was limited in opportunities for experience.”*

*“I enjoyed all OS clinics, just wish there was even more hands-on experience.”*

All of the students felt confident to diagnose and manage acute pericoronitis, most (95%) of them felt confident to manage haemorrhage from a socket, and all of them felt confident to assess an impacted mandibular third molar with respect to the guidelines and recognise the need for surgical removal. The majority (95.1%) felt confident to recognise the clinical features of potentially malignant and malignant lesions of the oral cavity and write an appropriate referral letter to a specialist in an appropriate time frame dependent on the clinical problem (92.7%). The majority (80%) felt competent to differentiate between pain of odontogenic and non-odontogenic origin.

Thirty-five students (85.4%) felt that the extension to their studies had been positive. The main reason for this, given in the free text responses, was that it improved their confidence by increased experience and learning opportunities, and as well as improving the staff–student relationships.

Examples:

*“Without question, we would not have been prepared as ‘safe beginners’ without the additional year due to the extended lack of clinical contact and the difficulty in performing many procedures when we did eventually return to clinic.”*

*“I feel the extra year gave me additional confidence that I am well prepared for beginning independent practice—I would not have had the same confidence without the additional year.”*

*“I would not have felt confident if I graduated last year. I have been given a lot of extra opportunities and learned a lot in this extra year.”*

*“Relationships between staff and students appeared to have benefited, felt like staff got to know students more.”*

Twenty-nine students (70.7%) felt that COVID-19 had affected their clinical performance, 23 (56.1%) thought it affected their stress levels, and 32 (78%) thought that COVID-19 affected their ability to experience the kind of clinical cases for their level of experience. Thirty-four students (82.9%) felt that COVID-19 affected their ability to experience cases due to the staff’s reluctance to allow this due to time constraints, and 28 (68.3%) felt it was because of staff shortage due to illness related to COVID-19. Overall, 11 (26.8%) of the students thought that the extension was detrimental for them mainly for financial or psychological reasons as shown in the free text responses.

Examples:

*“Financially and socially devastating. Declined mental state. The idea of being a dentist almost became disenchanted.”*

*“Severe financial implication for me. Negatively impacted my mental health as I was unhappy in my final year of studies here and just wanted to be done with it.”*

*“I would have gained a lot more experience and at a faster rate if I had of completed VT [Vocational Training] this year.”*

*“While it was a year of experience it was also a year not living my life the way I had planned.”*

### 3.2. Focus Group

Ten final year students, three males and seven females, responded to the email invitation to participate in an online focus group. The identity of the participants were known to the authors, but their responses are presented anonymously. The themes that emerged from the focus group session are represented under the broad headings of:

1. Experience during the extended final year;
2. Self-perceived confidence in exodontia and MOS;
3. Preparation for the next phase of their career;
4. The impact of COVID-19.

#### 3.2.1. Experience during the Extended Final Year

The OSB was generally well received, but the experience within each block could be very variable dependent upon the staffing, ill health, and patient attendance. The relative infrequency of the blocks meant that confidence tended to wane between the blocks, but this increased frequency would be welcomed by most of them. There was an appreciation that the students had greater numbers of blocks due to the extension. The participants suggested that the students could have flexibility within the OSB to ensure that all of them that were involved had a fair share of the surgical cases. However, it was acknowledged that this was very dependent upon the individual student’s personalities and professionalism, and it may not always work.

Examples:

*“The week block is a very good amount of time, maybe if they are increased in frequency if the timetable allows it. I know it’s difficult, but I think the week is a good amount of time.”*

Student 7

*“I think it very much depended on which week you came in and it varied quite a lot, some weeks you were very busy and were hands on doing things most session, but it does mean that you go for quite a long time between the hands-on session. It’s almost like you don’t get a chance to reinforce the learning you’ve had during that week because you haven’t done it again for another several months or so.”*

Student 6

*“I would just say I think that’s quite good, like having a run at it. But if that was available more than once a semester, it’d be good.”*

Student 1

*“Two times I didn’t have any MOS, so we just switched it. I mean it depends. It really depends on your group and what they’re like, but, If you’re willing to, you can just switch it around and it means that it’s a lot more fair.”*

Student 8

The variety of different clinics was well received such that attending the biopsy clinics might not allow them to gain much hands-on surgical experience, but it did offer opportunities to place sutures, while attendance in the consultant clinics did tend to focus their revision. The attendance in the extraction clinics (XLA) of 3rd year students was perceived not to be beneficial by some of them, but others felt it was good to reinforce their knowledge and skills by acting as a mentor to the junior students. Others felt it gave them an opportunity to develop leadership skills by facilitating the 3rd-year teaching.

Examples:

*“I think the biopsy clinics and one stop clinics were good because I think I did a lot of my oral medicine or revision on those as well, so I did find them really beneficial.”*

Student 9

*“I actually quite liked XLA, I ended up doing a tutorial with 3rd years going over different things. I think sometimes when you give someone the knowledge you have it’s a way of reiterating what you know. and I felt like it was quite a good learning experience as well.”*

Student 4

#### 3.2.2. Self-Perceived Confidence in Exodontia and MOS

Having targets for the number of extraction and MOS focused the need for experience, especially in the more reluctant students who may otherwise have chosen not to engage with MOS. Much of their extraction experience occurred in outreach facilities, and it exposed them to other staff who allowed them to use instrumentation that they may not have had experienced previously. The staff tended to allow them greater autonomy and freedom that they were accustomed to in the 3rd and 4th year, which was well received by the students who felt they had more confidence carrying out extractions, particularly while using adjuncts such as luxators.

Examples:

*“I got quite a lot of experience on XLA on outreach, it was mainly just getting the experience of MOS because obviously we needed our numbers.”*

Student 9

*“I feel as a final year student staff expected you to do more and they trusted you to do more so you felt more confident doing it and so the staff relationship was better.”*

Student 3

*“I think it depends how long after your last OSB it was, because when I did my last one, I was quite lucky and I got to do loads during that week. So if it was straight after that week I’d be like that. Yeah 100%. I would give it a shot and I think now I still would.”*

Student 2

Most of the students did feel confident in exodontia, but they did not feel confident to undertake surgery after graduation, with the exception of suturing. There was a generally feeling that they had adequate experience of intravenous sedation, with some students having provided treatment under sedation.

Examples:

*“I personally think independently I wouldn’t be able to do MOS, but if I had someone experienced behind me, and they were talking me through it, I think I would be able to.”*

Student 3

*“I did a couple of the surgicals under sedation and I also observed quite a number as well. So I don’t know if that’s just personal experience just by the luck of the draw, but I certainly saw several.”*

Student 4

#### 3.2.3. Preparation for the Next Phase of Their Career

Most of the students acknowledged that the extended year had been beneficial as it allowed them greater experience to prepare them to graduate and move into primary care. Some of them thought that the extension should only have been for one semester as they felt that time had been wasted.

Examples:

*“The transfer of what you read in a book into actually putting it into practice, you get a lot more confident with your clinical decision making. So I find that that aspect of dentistry I think strengthened quite a lot for me in the additional year.”*

Student 7

*“I think it was good to have the extra year, but I sometimes do think that Christmas graduation may have sufficed.”*

Student 4

The majority felt prepared for carrying out extractions in primary care, but most would not be happy to undertake surgery unless they had a trainer (students undergo a 1-year supportive training scheme in the UK with an experienced Vocational Trainer) who would be happy to support them. Many were worried about what to do if things went wrong.

Examples:

*“I definitely wouldn’t feel comfortable doing MOS, and it’s not from the teaching point of view. It’s more what if things go wrong that would concern me? It’s not the actual cutting, the flap and doing the procedure, it’s what if I do something wrong and I don’t feel like I’ve had enough advanced training to be able to defend myself.”*

Student 5

*“it’s not something I would be prepared to do without further training with the VT trainer and someone there to help me.”*

Student 6

#### 3.2.4. The Impact of COVID-19

Initially, there was a lot of stress and financial strain caused by the extension to their studies. Most of them felt that there were more positives than there were negatives from the extension to the course. The majority felt that the extension allowed for increased experience to be gained, which in turn improved their self-confidence.

Examples:

*“I just think that at the start it was everything was really scary. Then coming back after COVID you’d had such a long time of doing anything that it was like 2 steps backwards.”*

Student 4

*“I find that it was quite disheartening at the start and when we came back to the school it was quite slow, but when things picked up I know from my experience that talking to patients, management and communications got better.”*

Student 7

Many students acknowledged that the number of extraction and MOS where greater than they would have been achieved if they had graduated in 2021. At first, when they returned to the clinics, there were COVID-19-enforced changes with fewer patients and a reduced capacity due to the fallow time and social distancing measures. Other COVID-19 impacts were the illness among the staff and patients, causing clinic cancellations. Some students felt that the extension allowed the staff to get to know them more, and this fostered a better working and teaching environment, encouraging the students to obtain more opportunities and experiences.

Examples:

*“Quite often people were off with COVID or close contacts so it was quite difficult, I think you were kind of at the mercy of what the staffing might have been like that week.”*

Student 6

*“I felt coming out now, I probably have more confidence than I would have done from a normal fifth year just because between the 4th and coming into fifth year when COVID was kind of at its height and we’re just coming back onto clinic, we spent a lot of time in A&E because it’s one of the few clinics we were allowed on and we got that. I certainly got to do a lot more extractions.”*

Student 6

*“Yeah, from my point of view, I was not ready after the first final year to graduate. So I don’t grudge having done the extra year at all, because I think it was absolutely necessary.”*

Student 5

## 4. Comparisons with Other Final Year Experience and Self-Perceived Confidence in Oral Surgery

### 4.1. Comparison with Previous Final Year’s Data

The LiftUpp data for extractions and MOS were compared between the final year cohorts from 2017 to 2020 (Table 3). There was a significant difference between the years according to an ANOVA test. The post hoc tests showed that the mean number of extractions carried out by students in the extended year were significantly higher than they had been in all of the previous years, *p* < 0.001, as were the mean numbers of MOS, *p* < 0.001 (Table 4).

### 4.2. Correlations with National Data

Strong correlations were found between the extended year and the national survey [20] regarding their self-perceived confidence in surgery and knowledge in section B of the questionnaire (Table 5). A notable exception, which still shows no significative difference (*p* = 0.38), is the answer to Section B, Question 4c on the sectioning of teeth where the cohort of 2022 reported a lower confidence in sectioning teeth than those did in the national survey in 2009.

## 5. Discussion

Traditionally, at Dundee Dental School, the OS teaching for the final year students has been delivered in blocks to provide hands on experience of MOS, attendance at specialist clinics, and access to surgery under general anaesthetic. However, these weeks could not be standardised due to the staff leaving and variations in the timetabling and opportunities for clinical experience. The students have a target numbers of extractions to achieved before their final year, but they additionally have an opportunity to gain greater extraction experience in outreach clinics in their final year. They also have targets for MOS that they need to achieve by the end of 30 weeks of clinical teaching. Four students attend the OS department each week, allowing 1.5 weeks of teaching per student. Due to COVID-19, the teaching year of 2020-21 was significantly reduced, and even in the extended year of 2021-22, the COVID-19 restrictions still affected their clinical experience, with initially, there being only two surgical cases per list instead of three, reduced numbers of new patients on specialist clinics, the use of remote consultations, and the impact of staff and patients with COVID-19. Despite this, the extended year had greater opportunities to gain hands on experience than they had in pre-COVID-19 years as there were 46 weeks of clinical teaching.

Most of the students were positive that the OS course had prepared them for independent practice, and they felt confident to extract teeth and simple retained roots, but they felt less confident in aspects of surgery especially sectioning teeth. This finding agrees with those of other studies [20,23], and it suggests that there was comparably greater confidence than in one reported study [24]. This lack of confidence in surgery may reflect the type of cases available for student teaching, e.g., impacted third molars, which are referred to as secondary care, which are not ideal cases for novice operators especially if tooth sectioning is required. The confidence in suturing was high, but that skill is taught and assessed in the 4th year of the course with opportunities in that year to place sutures. The suggestion from the questionnaire and the focus groups was that more experience would have been desirable. However, as experience does not necessarily correlate with competence [25], a minimum realistic target was set for the students that they all achieved, and many of them exceeded it. Despite the improved experience gained in this extended year in comparison to that of the four pre-COVID-19 years, the students did not feel confident to perform surgery in a primary care environment without the support of a more experienced dentist, with the fear of litigation often being cited as the reason.

The confidence was high in the management of common conditions that present in primary care such as pericoronitis, the assessment of impacted third molars, the management of a haemorrhage, writing appropriate referral letters, and differentiating between pain of odontogenic origin, which is similar to previous findings [20,24]. Interestingly, this cohort seemed to report a greater level of confidence in the recognition and referral of potentially malignant lesions than they that which was reported in the previous studies [20,24,25,26].

The impact of the extension on the undergraduate dentistry students’ confidence in other disciplines and in performing other procedures is unknown. Although, minor oral surgical procedures are invasive they were consider droplet-spread rather than aerosol generating procedures and as such we were able to deliver this treatment and give student this experience early in the COVID recovery period. The comparisons with other disciplines such as restorative dentistry [27,28] or fixed prosthodontics [29] may prove to be enlightening.

Our findings suggests that the extension had a positive impact on the OS experience, and in turn, on the self-reported confidence in MOS and OS knowledge. In addition, the feedback from the focus group suggested that there were more general improvements in terms of better relations with the staff, improved communication, decision making, and treatment planning. Improved confidence has been reported as being directly related to clinical experience [23], but in contrast another study, the authors found no correlation between experience and confidence [26], and this may be because self-confidence in conducting surgery is multi-factorial [30,31].

However, the experience in conducting surgery despite, being it better than it had been in the pre-COVID-19 years, still falls short of that reported in other UK studies [15,26].

This research might suggest that there is only a very relationship weak linking the magnitude of work and competency measured as performance consistency. Quantitative recording systems which record the number of procedures fail to appreciate the true experience, and an inspection of other factors is required [32].

The student grade data are such that our students need to record a Grade of three or above (out of a 6-point scale) for the procedure to ‘count’ in terms of competence. As this assessment is clinical, with there being a wide range of presenting clinical cases with different levels of challenge, for example horizontal impacted wisdom tooth removal versus removal of a uncomplicated retained root, it is difficult for the teaching staff to firmly and definitively reconcile competence.

It has been demonstrated that a greater level of confidence follows training, and that this confidence is associated with observer-rated competence. This finding might support the notion that an assessment of self-report confidence is materially relevant when there is a real difficulty in measuring the standardised measure of competence, and that confidence might be seen as an indirect indicator of broad competence [33].

The negative impacts of the COVID-19 pandemic on the finances and mental health of the students cannot be underestimated, with many of the students commenting on how scared they felt not only in the clinical environment but also with the situation in general, which is in keeping with other studies [14,18,34]. However, the majority of them felt that the extension was necessary to prepare them to become a safe beginner and to prepare them for the next stage of their career. Some of them would have preferred an additional semester rather than a full year, but this was a minority proportion of them.

## 6. Limitations

The limitations of this study include the relatively poor response rate to the questionnaire, which is why a focus group was included to mitigate this and explore further themes. Despite this, the cohort is small, and it is not representative of the two Scottish dental schools who were affected by the COVID-19 extension to the final year. The small sample size makes comparisons with other studies with larger cohorts difficult, so our findings have to be interpreted with this caveat. Self-reported confidence does not correlate with competence, however, all of the students completed competency assessments or gateways for extractions and suturing, and they had to complete surgical cases to a satisfactory standard under staff supervision.

The uniqueness of this cohort is such that as they had an extra final year of study, it is not possible to readily replicate it. It may be possible to administer similar surveys to the other undergraduate student cohorts who were also required to undergo another extended year earlier in the curriculum before they became final year students. A further exploration of the current cohort regarding how they feel their careers have progressed since graduation (a survey of young dentists in practice) might be reveal the adequacy, or otherwise, of the teaching with the benefit of having real-world dental experience.

## 7. Conclusions

- The 1-year extension gave the students confidence in oral surgery clinical skills and knowledge in preparation for the transition to primary care and the next phase of their career.
- The impact of the extension caused significant anxiety and frustration amongst the final year student cohort who were affected.
- The students broadly accepted that without the extension, they would not have been adequately prepared for the next stage of their career.
- This cohort of final year students graduated with more OS experience than any of students from the previous 4 years from Dundee, which is comparable with those from other schools in the pre-COVID-19 era.

## Figures and Tables

**Table 1:** Focus group questions.

Number	Question
Q1	Did you think the block of one week (OSB) each semester was better than longitudinal session that you had in 3rd and 4th year?
Q2	What did you like the most about the OSB?
Q3	What did you like the least about the OSB?
Q4	What would you change about the OSB?
Q5	Did you think that you obtained enough hands-on experience of surgery? If no, why do you think that was the case?
Q6	Did you think going to operating theatres to observe was beneficial?
Q7	Do you think COVID-19 affected your experience in the OSB? Please explain.
Q8	Do you think the extension due to COVID-19 was necessary and do you understand the reasons why it was implemented?
Q9	Do you think the extension due to COVID-19 was beneficial?

**Table 2:** Students responses to Section B of questionnaire on self-perceived confidence (*n* = 41).

Section B Question	Likert Scale				
	SA	A	N	D	SD
(1) The teaching that I have received in oral surgery has given me sufficient knowledge to undertake independent practise.	14 34.1%	22 53.7%	4 9.8%	1 2.4%	0 0%
(2) I feel confident that I could extract an upper single-rooted tooth with an intact crown in an otherwise intact dentition.	34 82.9%	7 17.1%	0 0%	0 0%	0 0%
(3) I feel confident that I could remove visible retained roots of an upper left first molar using elevators or forceps.	21 51.2%	18 43.9%	1 2.4%	1 2.4%	0 0%
(4) I feel confident to assess and perform the surgical management of a failed extraction (e.g., a lower second molar) necessitating:					
(a) the raising of a mucoperiosteal flap;	1 2.4%	14 34.1%	14 34.1%	11 26.8%	1 2.4%
(b) bone removal;	1 2.4%	12 29.3%	10 24.4%	14 34.1%	4 9.8%
(c) sectioning the tooth to facilitate elevation of the roots;	0 0%	5 12.2%	14 34.1%	18 43.9%	4 9.8%
(d) wound closure using appropriate suture materials.	5 12.2%	33 80%	2 4.9%	1 2.4%	0 0%
(5) I feel confident to diagnose and manage acute pericoronitis.	33 80.5%	8 19.5%	0 0%	0 0%	0 0%
(6) I feel confident to manage haemorrhage from a socket.	25 61%	14 34.1%	1 2.4%	1 2.4%	0 0%
(7) I feel confident to assess an impacted mandibular third molar with respect to guidelines and recognise the need for surgical removal.	24 58.5%	17 41.5%	0 0%	0 0%	0 0%
(8) I feel confident that I can recognise the clinical features of potentially malignant and malignant lesions of the oral cavity.	18 43.9%	21 51.2%	2 4.9%	0 0%	0 0%
(9) I feel confident that I can write an appropriate referral letter to a specialist in an appropriate time frame dependent on the clinical problem.	13 31.7%	25 61%	1 2.4%	2 4.9%	0 0%
(10) I feel competent to differentiate between pain of odontogenic and non-odontogenic origin.	6 14.6%	27 65.9%	5 12.2%	3 7.3%	0 0%

Likert scale: SA = strongly agree; A= agree; N = neither agree nor disagree; D = disagree; SD = strongly disagree.

**Table 3:** Numbers of extractions and surgical procedures (MOS) for final year cohort of students in 2017–2022, given as the mean ± standard deviation.

Year	Numbers of Students	Extractions	MOS
2017	72	44.6 ± 11.5	2.5 ± 1.2
2018	65	43.0 ± 8.9	2.3 ± 1.0
2019	62	39.0 ± 6.3	2.4 ± 1.2
2020	62	41.7 ± 9.2	2.5 ± 1.0
2022	60	51.1 ± 9.7	3.3 ± 1.6

**Table 4:** Comparison between extraction and MOS between the extended year of 2022 and the previous years of 2017–2020 using a post hoc Least Significant Difference test.

Dependent Variable			Mean Difference	Std. Error	Sig.	95% Confidence Interval	
						Lower Bound	Upper Bound
extraction	2022	2017	6.56 *	1.644	0.000	3.32	9.79
		2018	8.18 *	1.703	0.000	4.83	11.53
		2019	12.17 *	1.683	0.000	8.86	15.49
		2020	9.423 *	1.703	0.000	6.07	12.77
MOS	2022	2017	0.781 *	0.214	0.000	0.36	1.20
		2018	0.993 *	0.222	0.000	0.55	1.43
		2019	0.937 *	0.219	0.000	0.49	1.36
		2020	0.767 *	0.222	0.001	0.33	1.20

* The mean difference is significant at the 0.05 level.

**Table 5:** Correlation between answers on self-perceived confidence in surgery from section B from the survey of final year students in 2009, *n* = 632 [20], and the Dundee University cohort of 2022, *n* = 41, using a Spearman’s correlation coefficient with a 95% confidence interval.

Question Number	Year	SA %	A %	NAD %	DA %	SDA %	*p* Value
B1	2009 2022	21 34	66 54	6 10	5 2	1 0	0.94
B2	2009 2022	77 83	22 17	1 0	0 0	0 0	1.00
B3	2009 2022	41 83	53 17	4 0	1 0	0 0	0.64
B4A	2009 2022	10 2	48 34	21 34	20 27	1 3	0.79
B4B	2009 2022	11 3	45 29	21 24	20 34	2 10	0.67
B4C	2009 2022	9 0	40 12	25 24	23 34	2 10	0.38
B4D	2009 2022	22 12	56 80	13 5	8 2	1 0	0.97
B5	2009 2022	48 80	48 20	2 0	1 0	0 0	0.79
B6	2009 2022	30 61	57 34	10 3	2 2	0 0	0.71
B7	2009 2022	37 58	54 42	7 0	1 0	0 0	0.89
B8	2009 2022	17 44	56 51	19 5	6 0	1 0	0.79
B9	2009 2022	20 32	59 61	13 2	6 5	0 0	0.95
B10	2009 2022	9 15	55 66	28 12	8 7	0 0	0.93
